# Prediction of pathologic prognostic factors in patients with lung adenocarcinomas: comparison of thin-section computed tomography and positron emission tomography/computed tomography

**DOI:** 10.1186/1470-7330-14-3

**Published:** 2014-04-22

**Authors:** Shingo Iwano, Mariko Kishimoto, Shinji Ito, Katsuhiko Kato, Rintaro Ito, Shinji Naganawa

**Affiliations:** 1 Nagoya University Graduate School of Medicine, Department of Radiology, 65 Tsurumai-cho, Showa-ku, Nagoya 466-8550, Japan; 2 Nagoya University Graduate School of Medicine, Department of Radiological and Medical Laboratory Sciences, 1-1-20 Daiko-Minami, Higashi-ku, Nagoya 461-8673, Japan

**Keywords:** Lung neoplasms, Adenocarcinoma, Computed tomography, Positron emission tomography, Fluorodeoxyglucose, Prognostic factor

## Abstract

**Background:**

The ratio of the maximum diameter of consolidation to the maximum tumor diameter (C/T ratio) on thin-section computed tomography (TSCT) and the maximum standardized uptake value (SUVmax) on ^18^ F-fluorodeoxyglucose positron emission tomography/computed tomography (PET/CT) are often used as preoperative independent variables to evaluate the invasiveness of lung adenocarcinoma. We investigated the associations between these independent variables and pathologic invasiveness in pulmonary adenocarcinomas.

**Methods:**

We selected patients with peripheral lung adenocarcinomas, definitively diagnosed by surgical resection, with diameters of ≤ 30 mm over a 4-year period ending in December 2010. The association between 3 independent variables (tumor size, SUVmax, and C/T ratio) and pathologic prognostic factors was evaluated using logistic analysis.

**Results:**

We evaluated a total of 163 primary lung adenocarcinomas in 148 patients (93 males and 55 females; age range: 34 to 84 years). Using multivariate logistic regression analysis, SUVmax and the C/T ratio were significantly associated with tumor invasiveness (odds ratio [OR] = 1.227; *p* = 0.025 and OR = 1.019; *p* = 0.008, respectively). Tumor size was not associated with invasiveness (OR = 1.003; *p* = 0.925). For solid type adenocarcinomas, only SUVmax was significantly associated with invasiveness (OR = 1.558; *p* = 0.003). For subsolid type adenocarcinomas, only the C/T ratio was significantly associated with invasiveness (OR = 1.030; *p* = 0.009).

**Conclusions:**

Both the C/T ratio and the SUVmax are significantly correlated with pathologic invasiveness in patients with small lung adenocarcinomas, while there was a difference between the 2 evaluations. Solid type adenocarcinomas with SUVmax values of ≥ 4.4 and subsolid type adenocarcinomas with C/T ratio ≥ 53% were so highly invasive.

## Background

Adenocarcinoma is the most common pathological type of peripheral lung cancer. However, even in patients with tumors of ≤ 3 cm in diameter, the postoperative prognosis varies, with poorly differentiated adenocarcinoma demonstrating a poor prognosis. Therefore, the use of preoperative imaging to determine tumor invasiveness is important, as limited resection (pulmonary segmentation or wide wedge resection) is currently recommended for non-invasive tumors [1-4].

Well-differentiated lung adenocarcinomas are often of a subsolid type, generally indicating a non-invasive nature and a good prognosis. These tumors may show ground-glass opacity on thin-section computed tomography (TSCT). On the other hand, poorly differentiated adenocarcinomas are usually of a solid type and have a tendency to be invasive, with a poor prognosis. These tumors consist entirely of consolidation on TSCT. This explains the usefulness of the C/T ratio, the maximum diameter of consolidation to the maximum tumor diameter on TSCT, in predicting the invasiveness and prognosis in patients with lung adenocarcinoma [1,3-7].

^18^F-fluorodeoxyglucose (FDG) positron emission tomography/computed tomography (PET/CT) is also essential in the preoperative evaluation of patients with primary lung cancer. The maximum standardized uptake value (SUVmax) of the primary lesion on PET/CT is well correlated with both tumor size and differentiation, with high-grade tumors tending to have a high SUVmax [8-10]. As with the C/T ratio, this value is used to determine tumor invasiveness and predict patient prognosis [11-18].

It is unclear whether the C/T ratio or the SUVmax value is a more suitable preoperative independent variables for predicting tumor invasiveness, or if there are any differences between them. We therefore conducted a retrospective investigation into the association between each of these independent variables, along with tumor size, and the pathologic invasiveness in patients with small (≤3 cm) pulmonary adenocarcinomas. In addition, we determined the most suitable independent variables cutoff levels to predict tumor invasiveness.

## Methods

This retrospective study was approved by the institutional review board (IRB) of our hospital. The IRB waived the need for written informed consent.

### Patient selection

We searched our institution’s records from November 2006 to December 2010 and selected patients who had preoperative PET/CT conducted for pulmonary tumors. We then obtained the clinical records, preoperative TSCT images, and postoperative pathological records for these patients. We limited our study to patients with peripheral lung adenocarcinomas of ≤ 30 mm in diameter that had been definitively diagnosed by pathological examination after surgical resection. We recorded the age, sex, and body weight of all patients, the pathological findings of the primary tumor (maximum diameter, lymph node metastasis, lymphatic permeation, vascular invasion, and pleural involvement) [17-20], and the pathological stage based on the Union for International Cancer Control guidelines, version 7.

### Preoperative TSCT

All preoperative TSCT examinations were performed using a 64- detector row scanner (Aquilion 64; Toshiba Medical Systems Corp., Tokyo, Japan). All scans were obtained from the lung apex to the diaphragm, during a breath-hold at deep inspiration, using the following parameters: x-ray tube voltage, 120 kVp; automatic tube-current maximum, 225 mAs; gantry rotation speed, 0.5 sec; and beam collimation, 64 × 0.5 mm. The TSCT images were reconstructed from 0.5-mm slices with intervals of 0.5 mm, using a high spatial-frequency reconstruction algorithm (FC52). No contrast medium was used.

### Measurement of consolidation/tumor ratio (C/T ratio)

**Figure 1:**
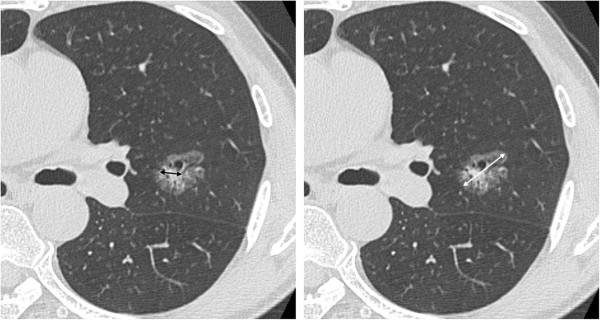
**Example of the consolidation/tumor (C/T) ratio measurement on thin-section computed tomography.** C/T ratio (%) = maximum diameter of consolidation (black arrow)/maximum diameter of tumor (white arrow) × 100.

Two radiologists independently evaluated the TSCT images using lung-window settings (window level, -600 Hounsfield units; window width, 1,800 Hounsfield units). The viewing monitor belonged to a picture archiving and communication system (RapideyeCore, Toshiba Medical Systems Corp., Tokyo, Japan). The radiologists calculated the C/T ratio, defined as the maximum diameter of consolidation over the maximum tumor diameter (Figure 1) [1,3-7]. The consolidation component was defined as an area of increased opacification that completely obscured the underlying vascular markings. Ground-glass opacity was defined as an area of a slight, homogenous increase in density that did not obscure the underlying vascular markings. Areas of ground-glass opacity often present as lepidic-predominant adenocarcinoma, typified by lepidic growth along alveoli, without invasive areas. Previous studies have shown that C/T ratio is related to aggressiveness and and prognosis in patients with lung adenocarcinoma. The mean value of the 2 observers for each tumor was used for the study analyses to reduce measuring error. We classified tumors into “solid type”, tumors containing no ground-glass opacities (C/T ratio = 100%), and “subsolid type”, tumors containing any ground-glass opacity (C/T ratio < 100%).

### Preoperative PET/CT

All preoperative PET/CT examinations were made using the Biograph 16 scanner (Siemens Medical Solutions, Erlangen, Germany). Patients were required to fast for at least 6 hours prior to imaging, and blood glucose levels were measured immediately prior to injecting FDG. Patients with blood glucose levels of >150 mg/dL were excluded from the study [3,17]. The FDG dose was determined by body weight, using either 3.7 MBq/kg (for patients weighing < 60 kg) or 4.07 MBq/kg (for patients weighing ≥ 60 kg). Fifty minutes after the intravenous injection of FDG, emission scans were acquired of the area between the proximal femora and the base of the skull; the 3-dimensional mode was used, with 1.7 min at each bed position. Breath-holding and respiratory gating were not used. PET images were reconstructed using the ordered-subset expectation maximization (OSEM) algorithm with 2 iterations and 16 subsets, incorporating a CT-based transmission map. No contrast medium was used for transmission CT according to the protocol of our institution.

### Measurement of maximum standardized uptake value (SUVmax)

The images were transferred to an e.soft Turbo-V workstation (Siemens Medical Solutions) where fusion PET/CT images were reconstructed. Regions of interest (ROIs) were placed around the pulmonary lesions; the maximum SUV value at a given ROI was defined as the SUVmax.

$$\begin{array}{l} \mathrm{SUV}=\mathrm{ROI}\;\mathrm{activity}\left(\mathrm{MBq}/\mathrm{mL}\right)\times \;\mathrm{body}\;\mathrm{weight}\left(g\right)\\ \;/\mathrm{injected}\;\mathrm{FDG}\;\mathrm{activity}\left(\mathrm{MBq}\right) \end{array}$$

The SUVmax represents the maximum glucose metabolic activity of the tumor.

### Statistical analysis

First, the associations between the 3 independent variables (tumor size, SUVmax, and C/T ratio) and the 3 pathologic prognostic factors (pleural involvement, lymphatic/vascular invasion, and lymph node metastasis) were compared using univariate logistic regression analysis. Pearson correlation coefficients between C/T ratio versus tumor size, SUVmax versus tumor size, and SUVmax versus C/T ratio were determined. Next, we defined invasive adenocarcinomas as tumors that had at least one invasive pathologic finding (pleural involvement, intratumoral vessel invasion, or lymph node metastasis) and non-invasive adenocarcinomas as tumors that had no invasive pathologic findings [6,7]. Multivariate logistic regression analysis was then used to identify independent predictors of non-invasive or invasive tumor nature, using the 6 independent variables (age, body weight, blood sugar level, tumor size, SUVmax, and C/T ratio) for all tumors, then separately for solid type tumors and subsolid type tumors. The mean value for each independent variables was compared, using unpaired Student’s t-test, between non-invasive tumors and invasive tumors. Patient characteristics (age, body weight, and blood sugar level) and tumor characteristics (size, SUVmax, and C/T ratio) were compared between the solid and subsolid groups using unpaired Student’s t-test, and the frequency of pathological invasiveness was compared between groups using the chi-square test. Receiver operating characteristic (ROC) curve analysis and Youden’s index were used to determine a cut-off level that would indicate invasiveness for SUVmax in solid type tumors and for the C/T ratio in subsolid type tumors [21]. Finally, recurrence rates within 3 years after surgery were compared using a Chi-square for independence test between non-invasive tumors and invasive tumors.

Excel 2007 (Microsoft Corp., Redmond, WA) and Statistical Package for the Social Sciences, version 21.0 (IBM Corp., Armonk, NY), were used to conduct all statistical analyses. A *p*-value of < 0.05 was considered significant. Pearson correlation coefficients of > 0.4 were considered as showing a positive correlation between variables.

## Results

### Study's cohort

**Figure 2:**
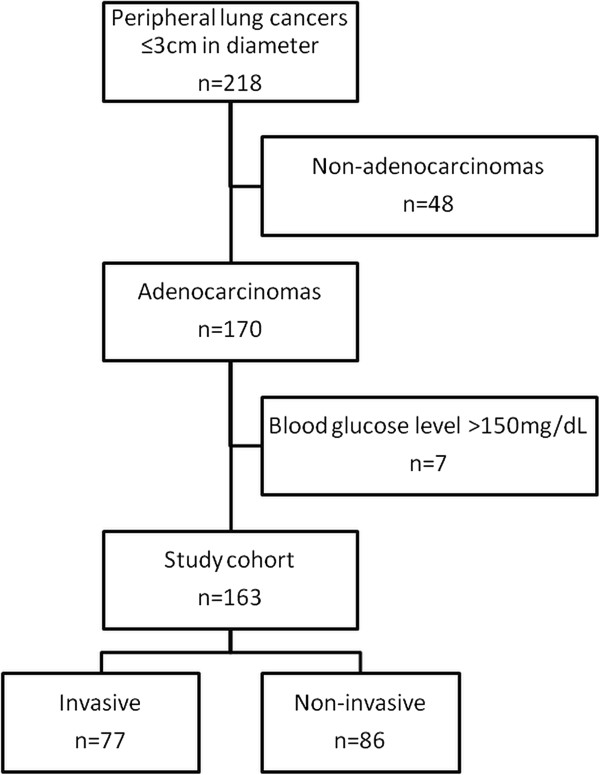
**Study cohort flow chart.** A total of 218 lung cancers of ≤ 30 mm in diameter were found from PET/CT and clinical records for a 4-year period. After exclusion, 163 adenocarcinomas were included in our analysis.

Figure 2 shows this study’s cohort flow chart. A total of 148 patients (93 male and 55 female) were evaluated, with an age range of 34 to 84 years and a median age of 68 years. These 148 patients had a total of 163 primary lung adenocarcinomas (13 patients had 2 simultaneous lesions and 1 patient had 3 lesions). There were a total of 51 lesions (31%) in the right upper lobe, 11 lesions (7%) in the right middle lobe, 37 lesions (23%) in the right lower lobe, 38 lesions (23%) in the left upper lobe, and 26 lesions (16%) in the left lower lobe. The median maximum lesion diameter was 19.0 mm. The mean ± standard deviation (SD) C/T ratio was 76.3 ± 33.3%, with a intra-class correlation coefficient between the 2 radiologists of 0.963 (95% confidential interval [CI] = 0.950 - 0.973, *p* < 0.001). Eighty-two lesions were of the solid type and 81 were of the subsolid type. The mean ± SD SUVmax was 2.8 ± 2.7. Ninety-two lesions (56%) had an SUVmax of < 2.5, and the other 71 lesions (44%) had an SUVmax of ≥ 2.5. The histological grade was G1 in 71 tumors, G2 in 76, G3 in 11, and GX (grade cannot be assessed) in 5 tumors. Pleural involvement was observed in 70 lesions, intratumoral vessel invasion (lymphatic and/or vascular invasion) in 24 lesions, and lymph node metastasis in 15 lesions. Sixteen lesions had 2 invasive pathologic findings (pleural involvement, intratumoral vessel invasion, or lymph node metastasis) and 8 lesions had all 3 invasive pathologic findings. Seventy-seven tumors (47%) were regarded as invasive; these had at least 1 invasive pathologic finding. The remaining 86 tumors (53%) were regarded as non-invasive. The pathological stage was stage I in 146 tumors, stage II in 8, stage III in 8, and stage IV in 1 tumor. The patient and tumor characteristics are summarized in Table 1.

**Table 1:** Patient and tumor characteristics

		**Mean**	**SD**	**Minimum**	**IQR**	**Maximum**
**Patients**						
	Age (years)	66	8	34	62 - 73	84
	Body weight (kg)	57	11	36	49 - 63.5	80
	Blood sugar (mg/dL)	93	14	61	84 - 101	141
**Tumors**						
	Size (mm)	18.8	6.5	4	13.5 - 24	30
	SUVmax	2.8	2.7	ND	0.9 - 4.1	14.1
	C/T ratio (%)	76.3	33.3	0	58.8 - 100	100

SD: standard deviation; IQR: interquartile range; SUVmax: maximum standardized uptake value; C/T ratio: consolidation diameter/tumor diameter ratio; ND: not detectable.

### Association between the independent variables and each pathologic prognostic factor

Table 2 shows the association between the independent variables and the pathologic prognostic factors using univariate logistic analysis. The tumor size was significantly correlated with pleural involvement and lymph node metastasis (*p* = 0.023 and *p* = 0.014, respectively), while size was not correlated with intratumoral vessel invasion (*p* = 0.158). The SUVmax was significantly correlated with intratumoral vessel invasion, pleural involvement, and lymph node metastasis (*p* < 0.001, *p* < 0.001, and *p* = 0.002, respectively). The C/T ratio was significantly correlated with intratumoral vessel invasion and pleural involvement (*p* = 0.014 and *p* < 0.001, respectively), but not with lymph node metastasis (*p* = 0.067). There was a positive correlation between SUVmax values and C/T ratios (Pearson correlation coefficient [r] = 0.48, *p* < 0.001). On the other hand, there was weak correlation between SUVmax and size (*r* = 0.36, *p* < 0.001); between C/T ratio and size (*r* = 0.20, p = 0.011).

**Table 2:** Univariate analyses for independent variables and pathologic prognostic factors

	**Pleural involvement**		**Intratumoral vessel invasion**		**Lymph node metastasis**	
	**OR (95% CI)**	***p*-value**	**OR (95% CI)**	***p*-value**	**OR (95% CI)**	***p*-value**
**Size**	1.060 (1.008-1.115)	0.023	1.052 (0.981-1.128)	0.158	1.130 (1.025-1.247)	0.014
**SUVmax**	1.290 (1.119-1.487)	< 0.001	1.379 (1.176-1.616)	< 0.001	1.293 (1.096-1.527)	0.002
**C/T ratio**	1.023 (1.010-1.035)	< 0.001	1.073 (1.014-1.135)	0.014	1.032 (0.998-1.067)	0.067

OR: odds ratio; CI: confidence interval; SUVmax: maximum standardized uptake value; C/T ratio: consolidation diameter/tumor diameter ratio.

### Association between the independent variables and the tumor invasiveness

Table 3 shows the association between independent variables and tumor invasiveness using multivariate logistic regression analysis. The SUVmax and C/T ratio were significantly correlated with tumor invasiveness (*p* = 0.025 and *p* = 0.008, respectively), while tumor size was not correlated with invasiveness (*p* = 0.925). For solid type adenocarcinomas, multivariate logistic analysis demonstrated that only SUVmax was significantly correlated with invasiveness (*p* = 0.009). Conversely, in subsolid type adenocarcinomas, only the C/T ratio was significantly correlated with invasiveness (*p* = 0.003). Table 4 shows the comparison between subsolid type and solid type adenocarcinomas. Solid type tumors had a significantly higher rate of pleural involvement and intratumoral vessel invasion than subsolid type tumors.

**Table 3:** Multivariate logistic regression analysis for independent variables and tumor invasiveness

	**All (n = 163)**		**Subsolid type (n = 81)**		**Solid type (n = 82)**	
	**OR (95% CI)**	***p*-value**	**OR (95% CI)**	***p*-value**	**OR (95% CI)**	***p*-value**
**Age**	1.033 (0.988-1.080)	0.158	0.982 (0.913-1.056)	0.627	1.069 (1.000-1.142)	0.050
**Body weight**	0.996 (0.963-1.029)	0.798	0.998 (0.950-1.048)	0.930	1.009 (0.957-1.063)	0.746
**Blood sugar**	0.982 (0.956-1.010)	0.202	0.952 (0.912-0.994)	0.024	1.027 (0.979-1.077)	0.279
**Size**	1.003 (0.944-1.066)	0.925	1.032 (0.939-1.135)	0.511	0.988 (0.899-1.086)	0.798
**SUVmax**	1.227 (1.026-1.469)	0.025	0.880 (0.636-1.217)	0.439	1.558 (1.157-2.099)	0.003
**C/T ratio**	1.019 (1.005-1.034)	0.008	1.030 (1.007-1.053)	0.009	NA	NA

OR: odds ratio; CI: confidence interval; SUVmax: maximum standardized uptake value; C/T ratio: consolidation diameter/tumor diameter ratio; NA: not available.

**Table 4:** Comparisons between solid type and subsolid type adenocarcinomas

	**Subsolid type (n = 81)**	**Solid type (n = 82)**	***p*-value**
**Age (years)**	66 ± 7	66 ± 9	0.951
**Body weight (kg)**	56 ± 10	59 ± 10	0.052
**Blood sugar (mg/dL)**	94 ± 16	92 ± 11	0.359
**Size (mm)**	19.0 ± 6.7	18.6 ± 6.2	0.737
**SUVmax**	1.7 ± 2.0	3.8 ± 2.9	< 0.001
**C/T ratio (%)**	52.3 ± 33.1	100.0 ± 0.0	< 0.001
**Pleural involvement (n)**	27 (33%)	43 (52%)	0.014
**Intratumor vessel invasion (n)**	4 (5%)	20 (24%)	< 0.001
**Lymph node metastasis (n)**	6 (7%)	9 (11%)	0.431
**Invasive tumor (n)**	28 (35%)	49 (60%)	0.001

SUVmax: maximum standardized uptake value; C/T ratio: consolidation diameter/tumor diameter ratio.

The invasive tumors demonstrated a significantly larger size than non-invasive tumors (*p* = 0.016), and higher values for SUVmax and the C/T ratio (*p* < 0.001 and *p* < 0.001, respectively) (Table 5).

**Table 5:** Comparisons between non-invasive and invasive adenocarcinomas

	**Non-invasive (n = 86)**	**Invasive (n = 77)**	***p*-value**
**Age (years)**	65 ± 8	67 ± 8	0.184
**Body weight (kg)**	56.8 ± 10.7	57.4 ± 10.3	0.704
**Blood sugar (mg/dL)**	95.7 ± 14.9	91.0 ± 12.5	0.031
**Size (mm)**	17.8 ± 6.7	20.0 ± 6.0	0.016
**SUVmax**	1.86 ± 1.80	3.80 ± 3.10	< 0.001
**C/T ratio (%)**	65.1 ± 37.7	88.9 ± 22.0	< 0.001

SUVmax: maximum standardized uptake value; C/T ratio: consolidation diameter/tumor diameter ratio.

Values are means ± standard deviations.

### A suitable cutoff value for determining invasiveness

In the analysis of solid type adenocarcinomas, the results of the ROC analysis for SUVmax showed an area under the curve (AUC) of 0.727 (95% CI = 0.618-0.836). A suitable cutoff value for determining invasiveness was estimated to be 4.4. This value yielded a sensitivity, specificity, positive predictive value (PPV), and negative predictive value (NPV) for tumor invasiveness of 55%, 88%, 84% and 55%, respectively. In the analysis of subsolid type adenocarcinomas, the results for the C/T ratio showed an AUC of 0.736 (95% CI = 0.618-0.852). A suitable cutoff value for determining invasiveness was estimated to be 53%. This value yielded a sensitivity, specificity, PPV, and NPV of 89%, 64%, 57% and 92%, respectively.

### Postoperative outcomes

Table 6 shows the postoperative outcomes for 125 patients with solitary lung adenocarcinomas. Twenty patients with simultaneous and metachronous multiple lung cancers were excluded from this analysis. Three patients were also excluded because they changed to another hospital within 3 years after surgery. Invasive adenocarcionomas showed a significantly higher recurrence rate than did non-invasive tumors (25% versus 6%, *p* = 0.004). Moreover, no local recurrence or distant metastases were found in 16 patients who had limited resections for non-invasive tumors. In contrast, a case of T1N0 invasive adenocarcinoma with intratumoral lymphatic permeation relapsed in a mediastinal lymph node after segmentectomy. This lesion was 13 mm in diameter, solid type (C/T ratio = 100%), and had a SUVmax of 4.8.

**Table 6:** Postoperative outcomes with solitary lung adenocarcinomas

		**Total (n)**	**Recurrence within 3 years (n)**	**Recurrence rate (%)**
**Invasive**		61	15	25
	Lobectomy	54	14	26
	Limited resection	7	1	14
**Non-invasive**		64	4	6
	Lobectomy	48	4	8
	Limited resection	16	0	0

Eight of 90 (9%) patients who had a SUVmax of <4.4 relapsed after surgery, while 15 of 35 (43%) patients who had a SUVmax of ≥ 4.4 relapsed. No local recurrence or distant metastases were found in 21 patients who had a C/T ratio <53%.

## Discussion

We were able to clarify several points in this study. First, both the C/T ratio (on TSCT) and the SUVmax (on PET/CT) are significantly correlated with invasiveness in patients with small adenocarcinomas. Next, an important difference exists between the C/T ratio and the SUVmax value: the C/T ratio is useful for predicting invasiveness only in subsolid type adenocarcinomas, while the SUVmax is suitable for solid type tumors. Finally, the cutoff C/T ratio for determining invasiveness in subsolid type adenocarcinomas is around 53%, while the cutoff for SUVmax in solid type adenocarcinomas is around 4.4.

The ability to identify prognostic pathologic factors on preoperative diagnostic imaging is essential to the determination of appropriate therapeutic strategies. Most small pulmonary adenocarcinomas are treated with pulmonary lobectomy, however, there has been a recent call to expand the indications for limited resection (e.g., segmentectomy or wide wedge resection) because of the advantage to the patient in preserving pulmonary function. Unfortunately, limited resection carries the risk of locoregional recurrence due to the possibility of inadequate surgical margins and incomplete lymph-node clearance [1,22-24]. Therefore, to reduce risk to patients, it is very important to be able to determine preoperatively which tumors are likely to be non-invasive [2-4]. In the present study, a case of adenocarcinoma with a high C/T ratio and a high SUVmax value relapsed after segmentectomy. Our results demonstrate that both TSCT and PET/CT findings may be useful in deciding which patients are better suited for limited resection.

We found that the SUVmax was significantly correlated with all pathologic prognostic factors; in other words, patients with higher SUVmax values have more highly invasive tumors. The C/T ratio was also significantly correlated with pleural involvement and intratumoral vessel invasion, but not with lymph node metastasis. A higher C/T ratio indicates that the tumor is associated with a greater amount of consolidation, and these tumors are more highly invasive. The reason that lymph node metastasis did not show an association with the C/T ratio in this study may have been because the absolute number of lymph node metastases was small (n = 15). In the present study, we selected lung adenocarcinomas of ≤ 3 cm in diameter, that is, T1a-b in the TNM staging system by reference to the study of Asamura et al. [4]. They reported a C/T ratio 0.25 or less in cT1a (≤2 cm) and 0.05 in cT1a-b (≤3 cm) were both able to define a homogenous group of patients with an excellent prognosis. In our study, tumor size is correlated with pleural involvement and lymph node metastasis, but not with intratumoral vessel invasion. Multivariate analysis showed that both the C/T ratio and the SUVmax value are independent and significant variables for predicting the invasiveness of lung adenocarcinoma, but tumor size is not significant. The C/T ratio and the SUVmax value may provide valuable information about tumor differentiation in small adenocarcinomas.

Next, we classified adenocarcinomas into solid type and subsolid type. The solid type tumors all have a C/T ratio of 100%, consisting only of consolidation, and therefore the C/T ratio cannot be used to predict invasiveness in this group. SUVmax is a significant independent variable for invasiveness in patients with solid type tumors, but only the C/T ratio is independently correlated with invasiveness in those with subsolid type tumors. We suggest, based on these results, that the C/T ratio should be evaluated first, and SUVmax should be used to predict invasiveness and prognosis only in patients with solid type tumors.

Finally, we evaluated suitable cutoff values for predicting invasiveness in both the solid- and subsolid types of tumors. The SUVmax cutoff is 4.4 in patients with solid type tumors. SUVmax is correlated with both tumor size and histopathologic characteristics, with well-differentiated adenocarcinomas demonstrating low SUVmax values. Thus, a SUVmax of < 4.4 may be helpful in determining that a solid type tumor is well-differentiated and non-invasive. However, the sensitivity for tumor invasiveness is only 55% at this cutoff value, meaning that PET/CT cannot absolutely exclude invasiveness. Therefore, an additional diagnostic method is necessary to increase the accuracy of this evaluation. It is important to note that adenocarcinomas with SUVmax values of ≥ 4.4 are so highly invasive that they are unsuitable for limited resection. In the study of Ohtsuka et al., patients with Stage I lung adenocarcinomas showing an SUVmax of ≥ 3.3 had poorer disease-free survival than those with an SUVmax <3.3, for patients with Stage I [14]. Turning our attention to subsolid tumors, the C/T ratio cutoff was found to be 53%. This is consistent with previous studies concluding that a C/T ratio < 50% is consistent with a non-invasive tumor [4,6,7]. As the sensitivity for tumor invasiveness is 89% at this cutoff, high C/T ratio tumors are unsuitable for limited resection.

This study has 3 limitations, the first of which is its retrospective and single-center nature. Second, we did not investigate histopathologic types other than adenocarcinoma; squamous cell carcinoma and large cell carcinoma are generally solid on TSCT and have a higher SUVmax than adenocarcinoma on PET/CT. Third, the PET/CT scans were acquired 50 minutes after the injection of FDG according to the protocol of our institution. This is somewhat early phase and may have influence on the value of SUVmax.

## Conclusions

In conclusion, both the C/T ratio on TSCT and the SUVmax value on PET/CT are significantly associated with the level of invasiveness in small adenocarcinomas. These measures are superior to tumor size for predicting tumor invasiveness. However, there is a difference between the 2 evaluations: SUVmax and C/T ratio were significantly associated with invasiveness of solid type adenocarcinoma and subsolid type adenocarcinoma, respectively. Thus, the C/T ratio should be evaluated first, and then SUVmax should be used to predict invasiveness only in patients with solid type tumors.

## Abbreviations

TSCT: Thin-section computed tomography; C/T ratio: Ratio of the maximum diameter of consolidation to the maximum tumor diameter; FDG: ^18^F-fluorodeoxyglucose; PET/CT: Positron emission tomography/computed tomography; SUVmax: Maximum standardized uptake value; OSEM: Ordered-subset expectation maximization; ROC: Receiver operating characteristic; AUC: Area under the curve; CI: Confidential interval.

## Competing interests

The authors declare that they have no competing interests.

## Authors’ contributions

SIw are responsible for the concept and design of the study. SIw drafted and wrote the paper. MK participated in the design of the study and helped to draft manuscript. MK, SIt, KK, and RI is contributed to the clinical data acquisition and reviewed the manuscript. SN contributed to critical revision of the manuscript. All authors read and approved the final manuscript.
